# Etiology-Specific Analysis of Hepatocellular Carcinoma Transcriptome Reveals Genetic Dysregulation in Pathways Implicated in Immunotherapy Efficacy

**DOI:** 10.3390/cancers11091273

**Published:** 2019-08-30

**Authors:** Wei Tse Li, Angela E. Zou, Christine O. Honda, Hao Zheng, Xiao Qi Wang, Tatiana Kisseleva, Eric Y. Chang, Weg M. Ongkeko

**Affiliations:** 1 Department of Surgery, University of California, San Diego, CA 92093, USA; 2 Department of Surgery, The University of Hong Kong, 21 Sassoon Road, Pokfulam, Hong Kong, China; 3 Department of Radiology, California and Radiology Service, VA San Diego Healthcare System, University of California, San Diego, CA 92093, USA

**Keywords:** cancer immunotherapy, TCGA, mutations, copy number variations, microRNAs

## Abstract

Immunotherapy has emerged in recent years as arguably the most effective treatment for advanced hepatocellular carcinoma (HCC), but the failure of a large percentage of patients to respond to immunotherapy remains as the ultimate obstacle to successful treatment. Etiology-associated dysregulation of immune-associated (IA) genes may be central to the development of this differential clinical response. We identified immune-associated genes potentially dysregulated by alcohol or viral hepatitis B in HCC and validated alcohol-induced dysregulations in vitro while using large-scale RNA-sequencing data from The Cancer Genome Atlas (TCGA). Thirty-four clinically relevant dysregulated IA genes were identified. We profiled the correlation of all genomic alterations in HCC patients to IA gene expression while using the information theory-based algorithm REVEALER to investigate the molecular mechanism for their dysregulation and explore the possibility of genome-based patient stratification. We also studied gene expression regulators and identified multiple microRNAs that were implicated in HCC pathogenesis that can potentially regulate these IA genes’ expression. Our study identified potential key pathways, including the IL-7 signaling pathway and TNFRSF4 (OX40)- NF-κB pathway, to target in immunotherapy treatments and presents microRNAs as promising therapeutic targets for dysregulated IA genes because of their extensive regulatory roles in the cancer immune landscape.

## 1. Introduction

Hepatocellular carcinoma (HCC) is the most prevalent class of liver cancer and the second leading cause of cancer-related mortality around the world [1]. Late diagnosis of HCC is common because of current limitations in diagnostic methods. Patients with late stages of HCC have five-year survival rates of less than 16% [2]. The most effective standard treatments of HCC, including liver transplantation, ablation, or surgical resection, are only recommended for early stages of HCC and they have high rates of recurrence [3]. Systemic treatments, such as chemotherapy, which are commonly used in other cancers, are relatively ineffective in HCC because of resistance to therapeutic agents and poor metabolism in cirrhotic livers, which contribute to the development of about 90% of HCC cases [4]. The extremely limited treatment options for advanced stage HCC patients led to great interest in recent advancements in cancer immunotherapy.

Most previous immunotherapy studies in HCC, including cytokine or antigen-based therapies, have failed to achieve adequate anti-cancer effects [5]. However, recent interests in immunotherapy were stimulated by the success of oncolytic viral gene therapy using JX-594 [6], the use of anti-Glypican-3 (anti-GPC3) antibodies to neutralize GPC3 antigens present on 80% of HCC cells [7], and CTLA-4 blockade clinical trials [8]. Unwanted immunogenicity and limited response rates remain significant challenges despite the variety of treatment strategies under investigation [5].

An understanding of the mechanisms through which gene regulation leads to evasion of tumor cells from immune recognition is important for the continual advancement of immunotherapy. microRNAs (miRNAs) are non-coding RNAs 18–25 nucleotides long that regulate critical cellular processes, such as development, division, and differentiation through mRNA silencing. miRNA dysregulation has been consistently documented across a large body of studies in human cancers since the recognition of their regulatory significance. miRNAs have also been documented to modulate the development of cells in both the innate and adaptive immune system, as well as regulate the release of cytokines and other proteins that induce key immune processes, such as inflammation [9].

In this study, we first identified immune associated (IA) genes that were dysregulated in alcohol-related and hepatitis B-related HCC and then evaluated their potential regulation by miRNAs identified to be dysregulated in HCC in our previous study [10]. Alcohol consumption and viral hepatitis infection are established independent risk factors for the development of HCC, and documented synergism exists between them [11]. A gene qualifies as IA in our study if it is involved in a pathway that regulates immune processes in either the innate or adaptive immune system. The relationship between dysregulation of IA genes and HCC development was explored through statistical correlations with patient survival, clinical variables, and the expression of commonly mutated genes in HCC. Notably, we identified the dysregulation of several genes that were reported to contribute to immunotherapy success or failure in melanoma. Further functional analysis of IA genes we identified, along with their association to etiology and genomic alterations, may reveal unique immune status stratifications of HCC patients, which can be targeted in immunotherapy to improve the clinical response rate. Finally, we illustrate that regulatory miRNAs of key IA genes in HCC may be therapeutically targeted as a novel treatment strategy or as a complement to existing immunotherapy treatments.

## 2. Results

### 2.1. Identification of Dysregulated Immune-Associated Genes in Etiology-Specific HCC and Correlation with Patient Survival

We downloaded liver HCC transcriptome data from The Cancer Genome Atlas (TCGA) database for a total of 371 patients and the adjacent normal samples from 48 of these patients. The patients were divided into four cohorts: alcohol drinkers with hepatitis B (*n* = 30), drinkers without hepatitis B (*n* = 34), nondrinkers with hepatitis B (*n* = V109), and nondrinkers without hepatitis B (*n* = 101). The adjacent normal samples were divided into two cohorts: samples from drinkers and samples from nondrinkers. The patient’s hepatitis infection status was not taken into consideration in normal cohorts due to evidence that the transformation of normal cells into HCC cells occurs at the same time as the integration of hepatitis B viral DNA into the host cell genome [12]; therefore, adjacent normal cells most likely do not have viral DNA. We expected our characterization of the landscape of IA gene dysregulation in tumor samples to also include genes that were expressed in immune cells of the tumor microenvironment due to the limited purity of TCGA tumor samples [13].

**Figure 1:**
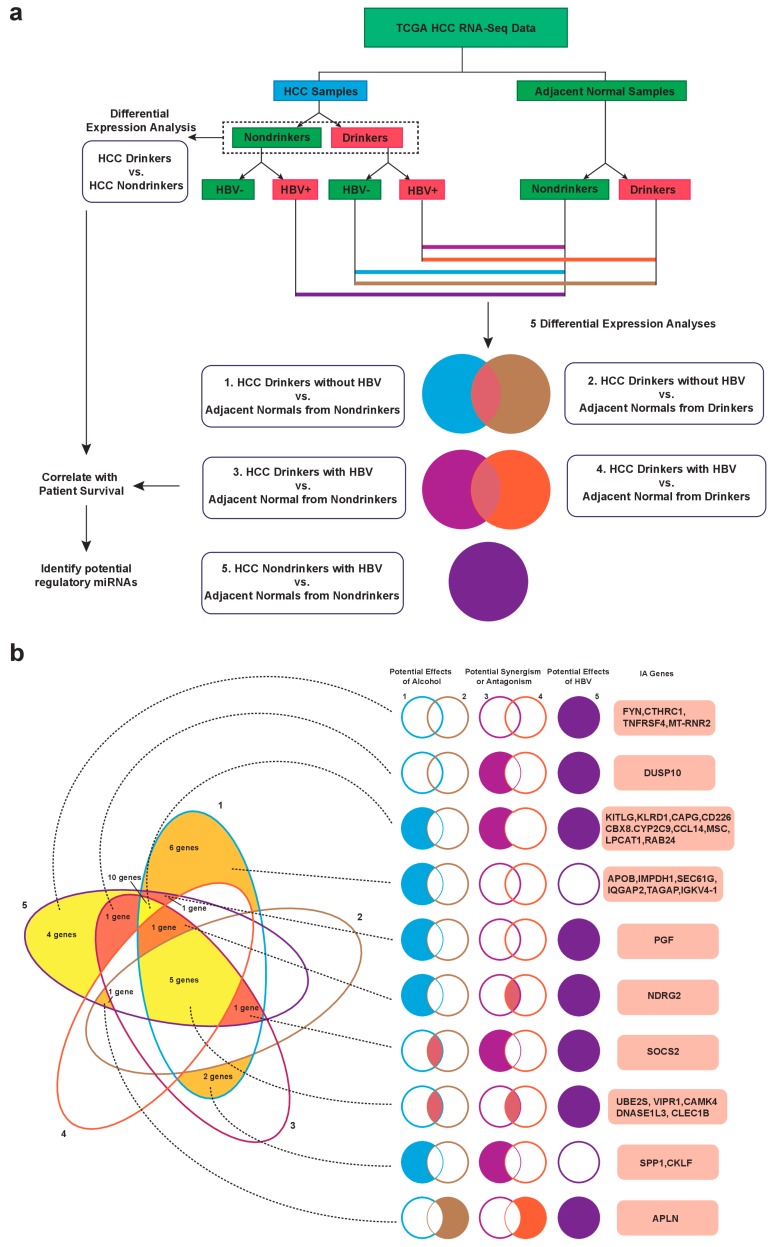

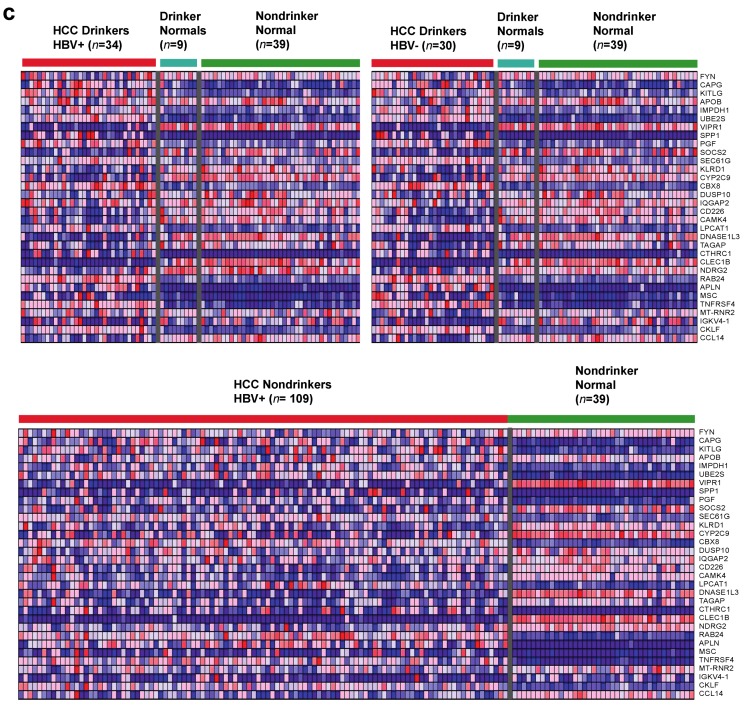
Summary of differential expression analyses results for identification of dysregulated immune-associated genes in hepatocellular carcinoma (HCC). (**a**) Schematic of workflow used to obtain the cohorts for six etiology-specific differential expression analyses is depicted. Each comparison is color-coded, with the color scheme consistent throughout (**a**,**b**). The five differential expression analyses comparing HCC samples to adjacent normal samples were divided into three sets. The first set includes the two comparisons involving samples from HCC drinkers without hepatitis B virus (HBV) and identifies immune associated (IA) genes potentially dysregulated as a result of alcohol consumption. The second set includes the two comparisons involving samples from HCC drinkers with HBV. Genes differentially expressed in this set can be used to examine possible synergism or antagonism between HBV-related HCC and alcohol-related HCC by comparing them to dysregulations identified in other sets of comparisons. The third set compares samples from HCC nondrinkers with HBV to normal samples from nondrinkers and identifies IA genes that were potentially dysregulated by HBV. (**b**) A five-set Venn diagram summarizes the number of IA genes identified as dysregulated in the five comparisons involving normal samples and any overlaps of genes between comparisons. The results are examined in terms of the three sets of comparisons described above. A solid color-filled region indicates the presence of differentially expressed genes for the indicated comparison(s). All IA genes presented correlate with patient survival data. (**c**) Three heatmaps are generated (one for each set of comparisons) for the thirty-two survival-correlated IA genes identified in the five HCC-normal comparisons. Refer to b for the genes differentially expressed in each individual comparison.

**Figure 2:**
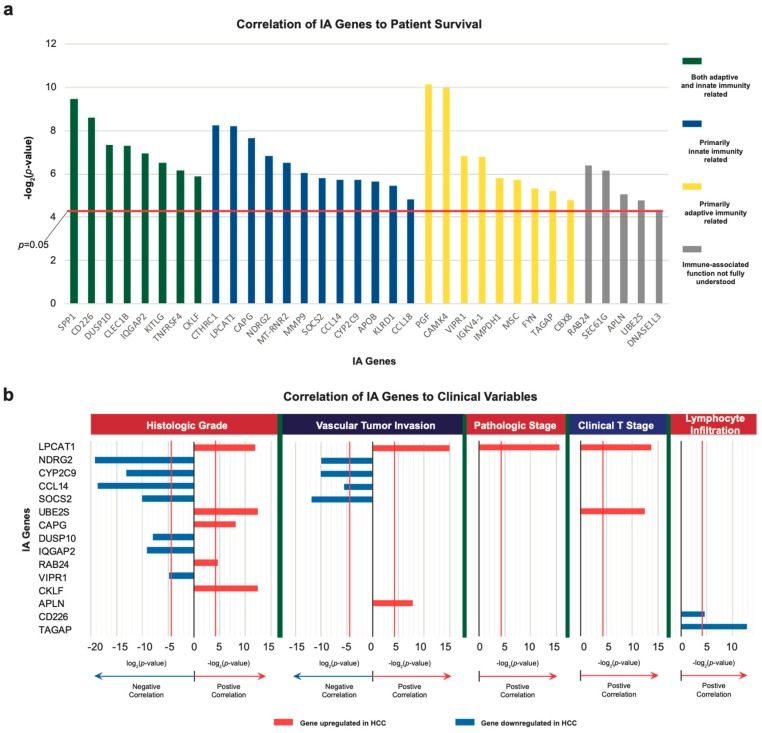

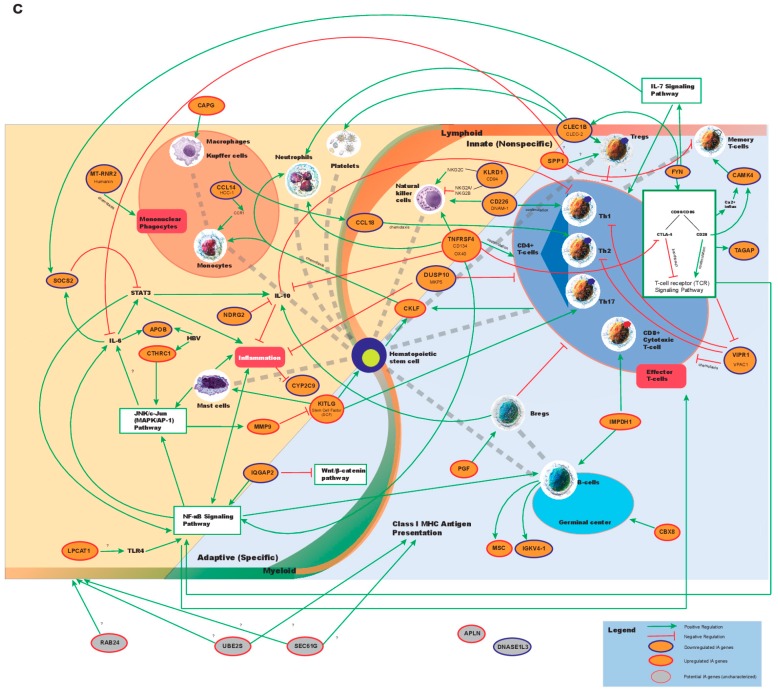
Correlation of IA gene expression with survival and clinical variables. (**a**) Bar graphs plotting the -log_2_(*p*-value) of correlation of IA gene expression with patient survival (Cox regression test, *p* < 0.05). (**b**) Bar graphs plotting the ± log_2_ (*p*-value) of correlation of IA gene expression with clinical variable (Kruskal-Wallis test, *p* < 0.05). Positive and negative correlation with variables are plotted in opposite directions on the graph. The greater the bars extend from 0, the higher the correlation between the variable and gene expression. (**c**) The interactions between dysregulated IA genes and key immune cells, processes, and pathways are mapped in this schematic. The graphical renderings of immune-cells were obtained from the galleries of Blausen Medical (https://en.wikiversity.org/wiki/WikiJournal_of_Medicine/Medical_gallery_of_Blausen_Medical_2014) and Concepts of Biology (http://philschatz.com/biology-concepts-book/contents/m45542.html).

A total of six differential expression analyses were performed to examine IA genes dysregulated in HCC cases with different etiologies (Figure 1a and Table 1). The expressions of differentially expressed genes identified were then correlated with patient survival data that were obtained from TCGA while using the Cox proportional hazards regression (*p* < 0.05, Figure 2a). Thirty-two survival-correlated IA genes were identified from the five differential expression analyses when comparing tumor vs. normal samples (Figure 1b). The probable etiology cause of gene dysregulation can be deduced from examining the overlaps and exclusions of differentially expressed genes that were found across different comparisons.

Six IA genes, *APOB*, *IMPDH1*, *SEC61G*, *IQGAP2*, *TAGAP*, and *IGKV4-1*, were dysregulated (differential expression, *p* < 0.05) in only tumor samples of drinkers without hepatitis B virus (HBV) infection as compared to pure normal samples (from nondrinkers). These six genes were not differentially expressed between the tumor samples from drinkers without HBV and normal samples from drinkers, which suggests that they are most likely exclusively dysregulated by alcohol. Four IA genes, *FYN*, *CTHRC1*, *TNFRSF4 (CD134)*, and *MT-RNR2*, are dysregulated in the samples from nondrinkers with HBV when compared to pure normal samples. These genes were not significantly dysregulated in any other comparison, which suggested that they are not genes that are essential to all malignant transformation and they are most likely exclusively dysregulated by HBV. Ten IA genes, *KITLG* (stem cell factor), *KLRD1*, *CCL14*, *CYP2C9*, *CD226*, *CBX8*, *LPCAT1*, *RAB24*, *CAPG*, and *MSC,* are dysregulated in the three comparisons of cancer samples with normal samples (HCC samples from drinkers with HBV infection vs. normal samples, HCC samples from drinkers without HBV vs. normal samples, and HCC samples from nondrinkers with HBV vs. normal samples). Following the above reasoning, we suggest that these genes are most likely dysregulated by both alcohol drinking and HBV infection independently, but they are not essential to malignant transformation. Two IA genes, *SPP1* and *CKLF*, are most likely dysregulated by alcohol, because they are differentially expressed in the same comparisons as those for the six alcohol-associated IA genes above. However, they are additionally dysregulated in tumor samples from patients who are both drinkers and HBV infected as compared to pure normal samples, which suggested a possibility for synergism of alcohol and HBV in dysregulating these genes. The dysregulation of two IA genes, *DUSP10* and *PGF*, seems to be antagonized by the interaction of alcohol and HBV. *DUSP10* is most likely dysregulated by HBV, but it does not appear to be dysregulated in a comparison between tumor samples from drinkers with HBV infection and normal samples from drinkers. *PGF* appears to be independently dysregulated by both alcohol and HBV, but it is not dysregulated in tumor samples from drinkers with HBV infection as compared to pure normal samples or to normal samples from drinkers. Some IA genes are likely to be central to the development of HCC tumor. These genes include *SOCS2*, *APLN*, *NDRG2*, and the five genes found to be dysregulated across all five cancer to normal sample comparisons: *VIPR1*, *CAMK4*, *CLEC1B*, *DNASE1L3*, and *UBE2S*. Figure 1b,c presents a complete summary of these results.

### 2.2. Correlation of IA Gene Expressions with Clinical Variables

To assess the clinical relevance and potential prognostic values of the IA genes that we identified, we correlated their expression to important clinical variables in HCC, including vascular tumor invasion, tumor histological grade, pathological and clinical stages of cancer, and lymphocyte infiltration percentage (Figure 2b). The IA genes, *LPCAT1*, *NDRG2*, *SOCS2*, *CCL14*, *UBE2S*, and *CYP2C9,* have expression levels that significantly correlate with two or more clinical variables, which suggests their potentially important role in contributing to disease progression.

To understand the relationship between the IA genes, we identified and their extent of involvement in immune-associated processes and summarized the known mechanisms of the functions for these genes in a schematic (Figure 2c).

### 2.3. Correlation of IA Gene Expressions with Copy Number Variations and Mutation Events Using REVEALER

**Figure 3:**
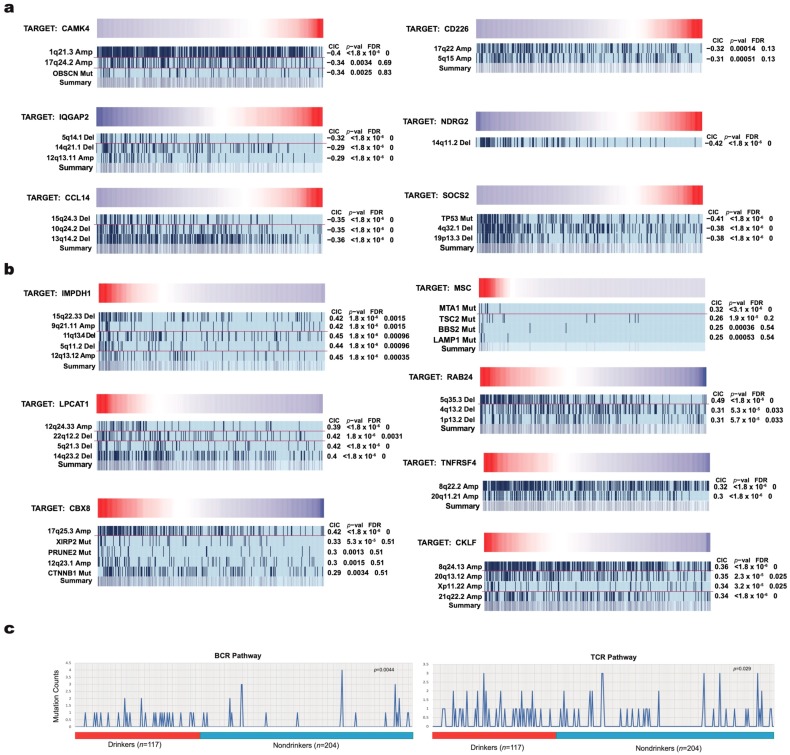
Correlation of IA gene expression to genomic alterations using REVEALER and differential mutation load in key immune-associated pathways. Genomic alterations are analyzed for correlation with low expression in (**a**) downregulated IA genes, while they are analyzed for correlation with high miRNA expression in (**b**) upregulated IA genes. The gradient bar displays the range of the IA gene expression, with the dark red extreme representing the highest expression and the dark blue extreme representing the lowest expression. Each patient sample would be assigned a specific spot along a light blue row based on expression of the IA gene within the sample, and if the indicated genomic alteration is present in a sample, it would be shaded as a dark blue bar. When many shaded bars are clustered towards an extremity of the row, it means many samples with very high or very low expression of the IA gene have the indicated genomic alteration, suggesting that the genomic alteration is significantly correlated with IA gene expression. Genomic alterations negatively correlated with IA gene expression result in negative CIC, while those positively correlated result in positive CIC. Caution should be taken when asserting correlations based on visual inspection because of the nonlinear distribution of expression values in each plot. Significant correlation was determined with CIC of around 0.30 or higher. A red dividing bar in between genomic features signifies a change in iteration. The Summary row combines results from multiple correlations to examine how well the set of genomic alterations identified could collectively account for gene dysregulation. (**c**) Line plots depicting differential mutation counts in B-cell receptor (BCR) and T-cell receptor (TCR) pathways for HCC drinkers versus HCC nondrinkers.

Copy number variations (CNVs) and mutations are widely recognized as key genomic alterations that drive cancer initiation and progression [14,15]. We used the REVEALER algorithm to systematically correlate all somatic CNVs and mutations that are present in each patient sample to IA gene expression in order to find a set of genomic alterations that are most likely responsible for the dysregulation of each IA gene. Given that genomic alterations initiate and sustain cancer, and that IA gene dysregulation is the likely cause for the sustenance of tumors against immune destruction, the alteration events that highly correlate with dysregulated IA gene expression in multiple patient samples are highly probable to be the cause of such dysregulation. Our REVEALER results illustrate that a large number of IA genes have significant correlation in expression with a set of genomic alterations, and there is minimal overlap between the genomic alterations that are implicated with each IA gene dysregulation (Figure 3a,b). This result is consistent with the diverse functions of these IA genes and the different IA pathways that they are involved in, as summarized in Figure 2c.

### 2.4. Identification of Frequently Disrupted Immune-Associated Pathways in HCC through Differential Mutational Load

Genetic mutations that are present in pathways regulating IA functions may reveal key insights into the mechanisms through which HCC evades the anti-cancer immune response. We compared the mutational load within the IA pathways of tumor samples from drinkers to those of tumor samples from nondrinkers to explore the effect of alcohol in dysregulating these pathways. We found that genes in the B cell receptor (BCR) signaling pathway and T cell receptor (TCR) signaling pathway have significantly higher rates of mutations in drinkers as compared to nondrinkers (fisher’s exact test—*p* < 0.05, Figure 3c and Table 2).

### 2.5. Identification of Potential miRNAs Involved in Regulation of IA Genes

We used the online target prediction service that was provided by TargetScan–release 7.1 to identify miRNAs that potentially target dysregulated IA genes identified in our study. The website was recently overhauled to incorporate a statistical model that was developed by Agarwal et al., who found their model to be significantly more accurate than other existing computational models for miRNA target prediction [16]. The list of potentially relevant miRNAs was filtered, so that only miRNAs that we identified in our previous study to be dysregulated in HCC in the opposite direction as the dysregulation of IA genes were retained as the candidates for further correlation.

**Figure 4:**
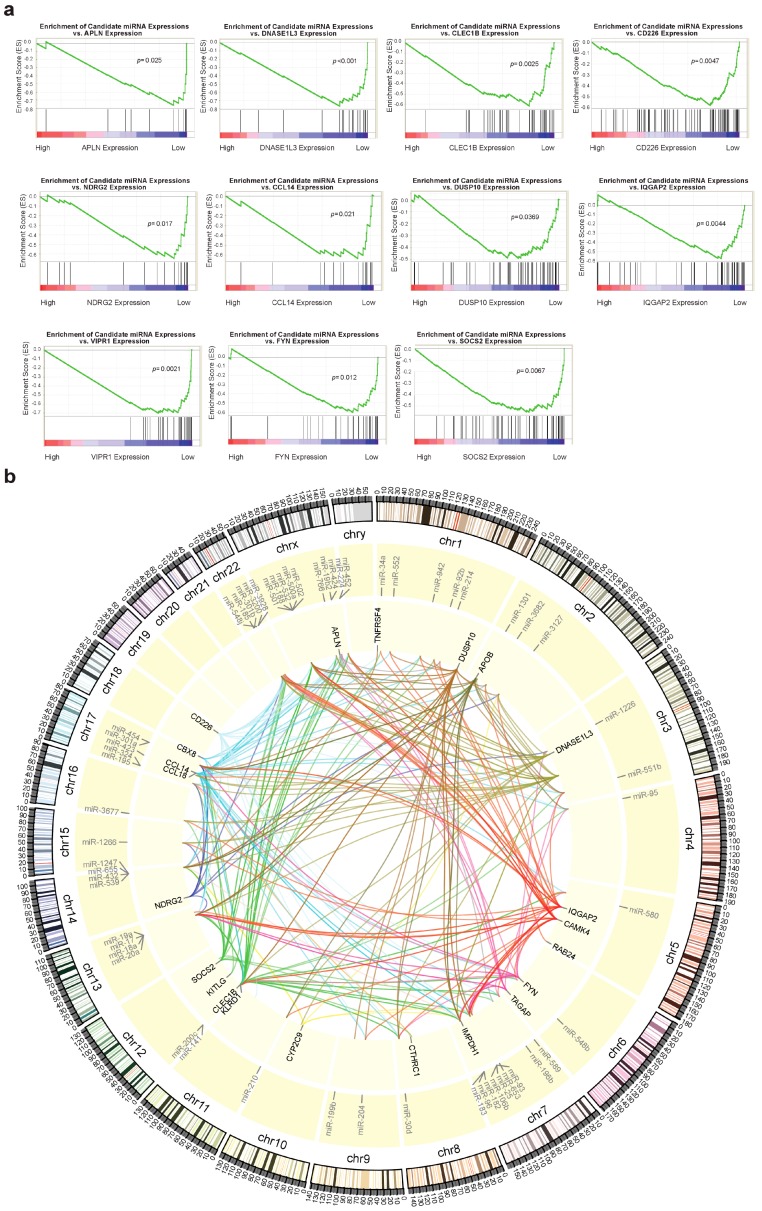
Identification of miRNAs dysregulated in HCC as potential regulators of dysregulated IA genes. (**a**) Gene set enrichment analysis (GSEA) plots (*p* < 0.05) correlate the expression of a set of miRNAs potentially targeting a given IA gene to its gene expression. Negative enrichment suggests that the expression of the miRNA set depresses the expression of the IA gene. (**b**) A Circos plot depicts the potential interactions between dysregulated miRNAs and dysregulated IA genes with their relative positions in the genome. Only miRNAs with three or more IA genes as potential targets are included in the plot. Potential interactions are defined as interactions forming the leading edge subsets of each GSEA plot. (See also Tables S1 and S2).

We used gene set enrichment analysis (GSEA) to investigate the correlation of miRNA expression with IA gene expression (Table S1). GSEA also allows for the quantification of the degree of synergistic gene expression suppression by multiple miRNAs (through enrichment score), as well as the ranking of miRNAs that were most closely associated with dysregulated IA genes (through rank metric score). The expression of a large number of miRNAs was found to be negatively enriched in relation to IA gene expression, which demonstrated an inverse relationship between miRNA expression and IA gene expression. Of the 34 dysregulated IA genes that we identified, 11 have 12 or more candidate regulatory miRNAs that potentially contribute to the suppression of gene expression (*p* < 0.05, Figure 4a). Four IA genes were not found to be targeted by any candidate miRNA. While only using miRNAs with core enrichment (as part of the leading-edge subset) and correlation with negative gene expression, we plotted the landscape of the potential interactions between candidate miRNAs and dysregulated IA genes in HCC (Figure 4b and Table S2).

### 2.6. In Vitro Validation of IA Gene Expression in Cell Lines after Alcohol Exposure

**Figure 5:**
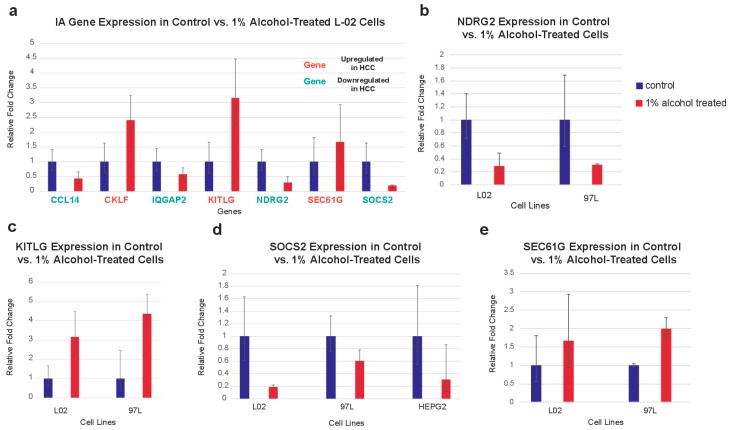
In vitro validation of IA gene dysregulation in liver cell lines by qPCR. (**a**) *CCL14*, *IQGAP2*, *NDRG2*, and *SOCS2* are downregulated, while *CKLF*, *KITLG*, and *SEC61G* are upregulated, after the L-02 cells are exposed to 1% alcohol. (**b**–**e**) *NDRG2* is downregulated, while *KITLG* and *SEC61G* are upregulated, in both MHCC-97L and L-02 cells after 1% alcohol treatment. *SOCS2* is downregulated in the HEPG2 cells, in addition to MHCC-97L and L-02 cells.

Quantitative PCR (qPCR) was used to measure the changes in expression of IA genes in cell lines after treatment with 1% ethanol. Only genes that were previously reported to be expressed in epithelial cells were examined. We found that, for the L-02 cell line, which was derived from normal human fetal hepatocytes, 6 IA genes dysregulated in our analysis are similarly dysregulated after alcohol exposure. The upregulated IA genes—*CKLF*, *KITLG*, and *SEC61G*—in our analysis are upregulated in alcohol-treated L-02 cells, while the downregulated IA genes *CCL14*, *IQGAP2*, *NDRG2*, and *SOCS2* are also downregulated in alcohol-treated L-02 cells (Figure 5a). *NDRG2*, *KITLG*, *SOCS2*, and *SEC61G* are also dysregulated in the HCC cell line MHCC97-L (Figure 5b–e). Additionally, *SOCS2* is downregulated in the HCC cell line HEPG2. We hypothesize that more IA genes are dysregulated in the normal liver cell line, because the HCC cell lines have already been transcriptionally reprogrammed by etiological factors.

## 3. Discussion

The current treatment options for advanced HCC have shown extremely limited efficacy. The only two drugs that are approved for treating advanced HCC in the United States, sorafenib and regorafenib, only lead to modest increase of the median survival rates of advanced HCC patients [17,18]. The partial response rate of sorafenib is only 2% [19]. Immunotherapy may be the key toward the effective treatment of advanced HCC. A phase I clinical trial with CTLA-4 checkpoint blockade has achieved 17.6% partial response rate in patients with advanced HCC [8]. Another clinical trial using the PD-1 checkpoint inhibitor nivolumab has reported 5% complete response and 18% partial response in advanced HCC [20]. A significant proportion of patients do not respond to immunotherapy treatment despite the promising potential of immunotherapy, particularly checkpoint blockades. When compared to sorafenib’s stable disease rate of 58.8%, PD-1 blockade performed worse, with 56% of patients (23 out of 41) in the clinical trial, discontinuing because of progressive disease [20]. The factors leading to the failure of immunotherapy in large fractions of patients remain poorly explored. Understanding differences in the immune status, including functional immune processes and their regulation, of different patients can shed light on the different observed clinical results. We systematically analyzed the gene expression data from TCGA to explore the landscape of dysregulated immune processes in HCC tumors and their surrounding environment. To the best of our knowledge, no previous study has identified the etiologic-specific dysregulation of IA genes or pathways in HCC through large-scale sequencing of RNA expression data, although Sia et al. examined the immune landscape of HCC and Thorsson et. al. examined the pan-cancer immune landscape of the TCGA samples [21,22]. We identified different IA genes that were dysregulated in HCC associated with hepatitis B virus (HBV) infection, attributed to 50% of HCC cases [23], and alcohol intake, which is also a well-established risk factor of HCC [24]. After exploring the relationship between IA gene dysregulation and miRNA dysregulation in HCC, we reveal that miRNA dysregulation might be the key contributor to differences in the immune status of HCC patients and that miRNAs serves as promising candidates for therapeutic intervention or profiling of patient immune status.

We observed several dysregulated genes in HCC that may be key contributors to the potential failure or success of PD-1 blockade, according to a recent study by Manguso et al. describing the effects of certain genes on immunotherapy outcome through loss-of-function screening [25]. Despite the many limitations of that study, including the screening of genes that were only expressed in a single melanoma cell line and the use of murine immune system as model, it was a significant study that sheds light on the many yet unknown factors that influence immunotherapy success. Genes in several pathways that were reported to sensitize tumors or cause resistance to immunotherapy were identified to be dysregulated in our study.

Manguso et al. reported that tumor cells lacking the gene *Jak1* are more resistant to PD-1 blockade [25]. JAK1 is critical to the function of the interferon-gamma signaling pathway and the IL-7 signaling pathway, which implies that reduced activity of these pathways may be responsible for resistance. Our study identified the downregulation of two genes participating in the IL-7 pathway: *FYN*, which codes for the tyrosine protein kinase Fyn and binds to the IL-7 receptor, forming a complex that JAK1 then binds [26]; and, *SOCS2*, the expression of which is induced by IL-7 signaling [27]. *FYN* is most likely downregulated by HBV infection according to our results, while the downregulation of *SOCS2* was observed in four out of five of our comparisons and may be non-etiology specific. The IL-7 signaling pathway is critical to the maintenance and survival of mature T-cells, and the downregulation of both *FYN* and *SOCS2* suggest a downregulation of IL-7 activity [28].

Inactivation of genes that are involved in the induction of the NF-κB pathway was reported to sensitize tumors to PD-1 blockade, which suggested that the activity of such genes may partly explain the failure of PD-1 based immunotherapy in certain patients [25]. We observed *OX40*, which is involved in the activation of canonical NF-κB signaling, to be upregulated in HBV-associated signaling. OX40, also known as TNFRSF4 or CD134, is a co-stimulatory receptor that is expressed on T-cells that binds to OX40L to target NF-κB1 [29]. Therefore, therapeutically antagonizing OX40 through the use of OX40 immunoglobulin may increase the response to PD-1 checkpoint inhibitors. Interestingly, OX40 is also known to increase T-cell viability and inhibit the CTLA-4 checkpoint molecule, so it can be theoretically engaged to complement CTLA-4 checkpoint blockade [30]. Because the wide range of pathways OX40 can potentially activate, further study of its functions in the context of immunotherapy will be useful. If OX40 complements immunotherapy in certain cases and induce resistance to immunotherapy in others, its expression levels in different patients may be a valuable criterion for selecting the appropriate immunotherapy drug to apply.

We also found one gene, *KLRD1* (*CD94*), dysregulated in a direction that potentially sensitizes tumor cells to PD-1 blockade, which may be a mechanism that contributes to the success of PD-1 blockade in certain HCC patients. Manguso et al. reported that the absence of Qa-1b (mice equivalent of HLA-E) binding to NKG2A, which is a inhibitory receptor, on T cells and NK cells increased the effectiveness of PD-1 blockade [25]. In humans, CD94 binds to NKG2A to form the CD94/NKG2A receptor, on which HLA-E then binds, in NK cells [31]. The gene *KLRD1* is likely to be downregulated by both alcohol drinking and HBV infection, which suggested decreased checkpoint activity in these HCC patients. Thus, patients with low *KLRD1* expression may be good candidates for the PD-1 blockade.

The limited results that were obtained by Manguso et al. represent one of the only sources of information elucidating the mechanisms contributing to differences in the effectiveness of immunotherapy. Therefore, we will summarize the rest of our findings in the context of their implications in the HCC immune landscape. The liver is evolutionarily highly tolerant of foreign antigens because of its exposure to blood containing microbial antigens and nutrients flowing from the intestines to the liver through the portal vein [32]. Immunosuppressive mechanisms include the upregulation of immune checkpoint molecules, an increase in the number of regulatory T cells, and the inhibition of natural killer cells [33]. Under this highly immunosuppressive environment, HCC tumor antigens can effectively evade immunity. We identified multiple IA genes dysregulated to increase immunosuppression. *CLEC1* (C-type lectin-like receptor 1B) is downregulated in all cancers as compared to normal tissue in differential expression comparisons, and codes for the protein CLEC-2, which is part of the same pathway as Fyn and is also critical in maintaining lymph node integrity [34,35]. *SPP1*, which is most likely upregulated by alcohol, codes for the protein osteopontin and it is expressed by both tumor cells and myeloid cells to mold an immunosuppressive tumor microenvironment [36]. Other dysregulations leading to immunosuppression include the upregulation of *PGF* and *CTHRC1* and downregulation of *IQGAP2*, *NDRG2*, *CCL14* (*HCC-1*), *CD226* (*DNAM-1*), and *CAMK4*.

On the other hand, HCC is also an inflammation-associated cancer, hence the tumor environment has potential immunogenicity [37]. Inflammation has been recognized as an important factor in malignant transformation and it results in the recruitment of large amounts of immune-associated cells into the tumor environment, which leads to the release of cytokines and growth factors to promote cellular proliferation and regeneration in response to necrosis in the tumor core [38]. We identified a number of IA genes dysregulated to increase immunogenicity. *VIPR1* (vasoactive intestinal polypeptide receptor 1, or *VPAC1*) is consistently downregulated across all cancer to normal sample comparisons. It is expressed on T-cells as a part of the VIP signaling axis, being responsible for suppressing cytokine production and increasing the number of inducible regulatory T-cells [39]. *DUSP10*, which is also known as *MKP5* (mitogen-activated protein kinase phosphatase 5), was identified to be likely downregulated by HBV infection in this study. The downregulation of *DUSP10* was found to increase the levels of pro-inflammatory cytokines and level of T-cell activation [40]. Other dysregulations leading to immunogenicity include the upregulation of *IMPDH1*, *KITLG* (stem cell factor), and *CKLF*.

Correlating the expressions of IA genes we identified with clinical variables allows for us to explore their clinical significance and importance in HCC pathogenesis and progression. Vascular tumor invasion is a strong prognostic factor in HCC and it is arguably the strongest predictor of recurrence after surgical resection or liver transplant [41]. The expressions of six IA genes exhibit significant statistical correlation with vascular tumor invasion. The expressions of several IA genes also correlated with tumor histological grade, pathological stage, and size of primary tumor (clinical T stage). Two IA genes, *CD226* and *TAGAP*, have expressions that directly correlate with lymphocyte infiltration, which suggest that increasing their expression may lead to clinically observable immune activation.

While using qPCR, we were able to validate the dysregulation of *NDRG2*, *KITLG*, *SOCS2*, and *SEC61G* in multiple liver cell lines after exposure to alcohol. In the normal liver cell line, L-02, we observed the dysregulation of *CCL14*, *CKLF*, and *IQGAP2,* in addition to the dysregulation of the genes above. *SEC61G* and *IQGAP2* are exclusively dysregulated by alcohol, while others are dysregulated in both HBV and alcohol-induced HCC, according to our analysis. Several of these genes, including *KITLG*, *CCL14*, and *CKLF*, are cytokines, which possibly provide a mechanism for gene dysregulation in tumor cells to affect the immune phenotype. Additionally, *NDRG2* regulates the release of cytokines in HCC cells, while SEC61G potentially mediates antigen presentation [42,43]. On the other hand, SOCS2 and IQGAP2 relay interactions with factors that are released by immune cells, including various types of cytokines, potentially allowing for cancer cells to be aware of the immune environment [44,45].

In search for potential causal genomic alterations that lead to the dysregulation of these IA genes, we applied the REVEALER algorithm to systematically correlate all mutations and CNVs that are present in HCC to IA gene expression. A diverse set of genomic alterations seems to be implicated in IA gene dysregulation, although we discovered that a number of commonly mutated genes in HCC, including *TP53*, *CTNNB1*, *XIRP2*, and *PRUNE2*, strongly correlated with the dysregulation of certain genes. In addition to common mutations, an increase in the frequency of mutations in IA pathways may offer an explanation to the dysregulation of IA genes. We found that the T-cell receptor pathway and B-cell receptor pathway both have higher mutational load in drinkers with HCC than in nondrinkers with HCC.

We chose the REVEALER correlation method to explore the critical application of our data: the stratification of patients into clinically relevant cohorts that differently respond to immunotherapy drugs, in order to select the most effective treatment option for each patient. REVEALER selects the genomic alteration with the strongest correlations as a seed for subsequent iterations, then the algorithm penalizes the CIC for genomic alterations that are present in patients who also possess the seed alteration, since the dysregulation of IA genes in those patients would be better accounted for by the seed [46]. This innovation allows for the stratification of patient immune status, which we sought to accomplish to some degree through examining the role of etiology in dysregulating IA genes. However, stratification using gene expression has limited effectiveness, because of deficiencies in the RNA sample quality, irreproducibility of expression signatures across different cohorts, and different compositions of cell populations in different samples [47]. An effective stratification method may be developed with genomic alterations when the effects of dysregulated IA genes on immunotherapy outcome, and the genomic alterations that are responsible for their dysregulation, are known. We observed that our REVEALER data adequately demonstrate REVEALER’s ability to identify multiple genomic alterations that are associated with each IA gene dysregulation and ensure that as diverse a set of patients as possible possesses these alterations.

We turned to microRNAs (miRNAs) to explore the potential treatment options for a complex stratified patient population. A single miRNA can target multiple mRNAs transcribed from genes in a single network [48]; therefore, therapeutically adjusting the expression of one miRNA can potentially reverse the dysregulation of a large number of genes. For example, the sensitivity of T cell receptors (TCRs) can be effectively increased by miR-181a, which increased interleukin production, stimulated T-cell proliferation, repressed antagonistic phosphatases, and also decreased the number of CTLA-4 molecules [49].

The limitations of previous studies of miRNA-mRNA regulation include an intense focus on validating or predicting individual miRNA-mRNA interaction and the consequent failure to examine complex miRNA-mRNA system interactions. We mapped the association of all dysregulated IA genes in HCC with all potential regulatory miRNAs dysregulated in HCC to assess the wide range of possible interactions that are available for therapeutic targeting.

From our correlations of miRNA dysregulation with IA gene dysregulation, we identified several miRNAs, including miR-106b, miR-17, miR-183, miR-20a, miR-25, miR-301a, miR-30d, miR-532, and miR-93, which can potentially regulate eight to ten different downregulated IA genes. The therapeutic inhibition of the expression of these miRNAs may lead to a dramatic improvement in the immune system’s ability to detect tumors. With the exception of miR-532, the upregulation of all the miRNAs that are listed above has been previously described as the prognostic factors of HCC or mechanistically linked to HCC development [50,51,52,53,54]. For example, miR-17 has been reported to regulate the IL-7 signaling pathway through targeting *JAK1* mRNA [55]. The dysregulation of these miRNAs may also be responsible for the HCC tumor evasion of immune processes by dysregulating a large number of IA genes.

## 4. Materials and Methods

### 4.1. RNA-Sequencing Datasets and Clinical Data

Level 3 normalized mRNA expression read counts for tumor samples from 371 hepatocellular carcinoma patients and patient clinical data were downloaded on 4 July 2017 from The Cancer Genome Atlas (TCGA) (https://tcga-data.nci.nih.gov/tcga). The mRNA read counts for adjacent solid normal tissue samples of 48 hepatocellular carcinoma patients were also obtained.

### 4.2. mRNA Differential Expression Analyses

mRNA read count tables were imported into edgeR v3.5 (http://www.bioconductor.org/packages/release/bioc/html/edgeR.html), and lowly expressed mRNAs (counts-per-million < 1 in an amount of samples that ware greater than the size of the smaller cohort of each analysis) were filtered from the analysis. Following TMM (trimmed mean of M-values) normalization, pairwise designs were applied to identify significantly differentially expressed mRNAs in (1) tumor tissue from HCC patients who are drinkers without HBV versus adjacent normal tissue from patients who are nondrinkers, (2) tumor tissue from HCC patients who are drinkers without HBV versus adjacent normal tissue from patients who are drinkers, (3) tumor tissue from HCC patients with HBV who are also drinkers versus normal tissue from patients who are nondrinkers, (4) tumor tissue from HCC patients with HBV who are also drinkers versus normal tissue from patients who are drinkers, (5) tumor tissue from HCC patients with HBV who do not drink versus normal patients from patients who do not drink, and (6) tumor tissue from HCC patients who drink versus tumor tissue from HCC patients who do not drink. Immune-associated genes, from which differentially expressed mRNAs were transcribed, were identified as dysregulated and retained as candidates. Differential expression is defined as *p* < 0.05 and fold change <−2 or >2 in edgeR analysis.

### 4.3. Association of Candidate Genes’ Expressions with Patient Survival and Clinical Variables

Survival analyses were performed while using the Kaplan–Meier Model, with gene expression being designated as a binary variable based on expression above or below the median expression of all the samples. Univariate Cox regression analysis was used to identify candidates that were significantly associated with patient survival (*p* < 0.05). Survival-correlated genes were evaluated for clinical significance. Employing the Kruskal–Wallis test, we investigated gene association with neoplasm histological grade, clinical and pathologic stages, vascular invasion of tumor, and percent lymphocyte infiltration while using clinical data and mRNA expression values (counts-per-million) from HCC patients. In clinical T stage analysis, patients with stages T1a and T1b were grouped into stage T1, and likewise for stages T2, T3, and T4.

### 4.4. Information-Coefficient Based Correlation of IA Gene Expression with Genomic Alterations

Mutation and copy number variation (CNV) data for the HCC tumors were obtained from mutation and CNV annotation files that were generated by the Broad Institute GDAC Firehose on 28 January 2016. Annotation files were compiled into a binary input file for the program REVEALER (repeated evaluation of variables conditional entropy and redundancy), which was designed to computationally identify a set of specific copy number variations and mutations that were most likely responsible for the change in activity of a target profile [46]. The target profile was defined in our study to be IA gene expression. REVEALER runs multiple iterations of the correlation algorithm, with the genomic feature exhibiting the strongest correlation in each run serving as a seed for the successive run to identify a set of most relevant genomic alterations. We set the maximum number of iterations to three. A seed is a particular mutation or copy number gain or loss event that most likely accounts for the target activity. When given a seed, REVEALER will focus correlation only on patients with altered target activity that was not accounted for by the seed. We set the seed to null for the first iteration. We set the threshold of genomic features to input to features present in less than 75% of all samples.

### 4.5. Identification of Differential Mutational Load in Immune-Associated Pathways

Immune-associated pathways were manually identified through gene sets described in existing literature. The number of genes with mutations in each immune-associated pathway was tallied for each patient tumor sample, and the Fisher’s exact test was performed to identify significant differential mutational load (*p* < 0.05) of each pathway in the samples from alcohol drinkers versus samples from nondrinkers.

### 4.6. Assessing Potential Involvement of miRNAs in Regulating IA Genes

To identify the possible regulatory miRNAs that were associated with IA genes, we identified a list of miRNAs predicted to bind to each dysregulated mRNA using TargetScan version 7.1 (http://www.targetscan.org/vert_71/) [16]. This list is then filtered to exclude any miRNAs not identified as dysregulated in HCC in our previous study [10], and only miRNAs that were dysregulated in a direction consistent with their regulatory roles of IA genes (i.e., miRNAs that were upregulated if the IA gene was downregulated) were retained as candidates.

The gene set enrichment analysis (GSEA) software was used to characterize the enrichment of miRNA expressions with respect to IA gene expressions [56]. The full set of candidate miRNAs for each IA gene was modeled as a gene set. The continuous expression values of IA genes were used as phenotype labels. The unfiltered expression values of all miRNAs available from TCGA miRNA expression datasets (*n* = 1535) were included in the expression dataset input file. One GSEA plot was produced for each IA gene that was potentially associated with seven or more candidate miRNAs.

### 4.7. Validation of IA Gene Expression with Quantitative PCR (qPCR)

The cultured cells were treated with 1% ethanol for 24 h. Specifically, 20 µL of pure ethanol was added to a culture plate with 2 mL of media. The plates were sealed following ethanol exposure to keep the ethanol from escaping and to maintain constant ethanol levels. Total cell lysate was collected and mRNA was extracted while using the RNeasy kit (QIAGEN). cDNA was then synthesized from 1.5 μg of total mRNA using reverse transcriptase (RT) (Invitrogen, Carlsbad, CA, USA), as per the manufacturer’s instructions. Real-time qPCR was performed by combining 2.5 μL of RT with 22.5 μL of SYBR green (Roche, Basel, Switzerland). The reaction was run while using System 7300 (Applied Biosystems, Foster City, CA, USA) and the results were analyzed by the relative quantity method. Experiments were performed in triplicates with GAPDH expression as the endogenous control. GAPDH was chosen as control, because it was not differentially expressed between the samples from drinkers vs. those from nondrinkers (*p* = 0.57), which suggested that alcohol does not alter GAPDH expression. Primers were custom designed by the authors and created by Eurofin Genomics, Louisville, KY, USA. The following sequences were used:

CCL14 forward: AATACAGCTAAAGTTGGTGGGG
CCL14 reverse: TCAAAGCAGGGAAGCTCCAA
CKLF forward: GGCACTAACTGTGACATCTATGA
CKLF reverse: TCACAAGTGCAAACACAAGCA
IQGAP2 forward: TCAAGTGTAGGAAGGAGTTGTGG
IQGAP2 reverse: CTGGATCTGGGGTGCTATTCC
KITLG forward: TATGTCCCCGGGATGGATGT
KITLG reverse: TTTGGCCTTCCCTTTCTCAGG
NDRG2 forward: GGGACAGGGATGGAAAATGGT
NDRG2 reverse: CCACATGAACCCGCACAAAG
SEC61G forward: TTTAGGTGTCGGTTGGGTAGG
SEC61G reverse: CTCACACCCTCACACTTGTTC
SOCS2 forward: AGAGCCGGAGAGTCTGGTTT
SOCS2 reverse: ATAGCGATCCTTGGCCCTTG

## 5. Conclusions

Our study demonstrated significant differences in the clinically relevant IA gene dysregulation landscape in HBV-induced and alcohol-induced HCC. We found that several dysregulated IA genes that are associated with pathways reported to contribute to immunotherapy effectiveness or resistance and identified several other dysregulation IA genes that we hope will be examined in the context of immunotherapy outcome in future studies. We correlated IA gene dysregulation to genomic alterations to explore potential methods of stratifying patients into clinically relevant populations because of the diverse expression profiles possible for different patients. Finally, we proposed a novel focus for HCC immunotherapy by examining dysregulated miRNA as the potential targets for therapeutic intervention of a stratified patient population. The presence of large numbers of dysregulated genes in the HCC immune landscape and differences in this dysregulation profile based on HCC etiology precipitate the importance of using regulatory molecules, such as miRNAs, as treatment targets to improve the patient response rate to immunotherapy.

## Figures and Tables

**Table 1:** Differential expression analysis results for survival-correlated immune-associated genes.

**HCC Drinkers without HBV vs. Nondrinker Normals**				**HCC Drinkers without HBV vs. Drinker Normals**			
**Gene Name**	**Fold Change**	**FDR**	***p*** **-Value**	**Gene Name**	**Fold Change**	**FDR**	***p*** **-Value**
APOB	−2.16	9.0 × 10^−13^	7.0 × 10^−10^	APLN	16.65	1.7 × 10^−6^	3.5 × 10^−4^
CAMK4	−4.12	7.2 × 10^−16^	3.1 × 10^−13^	CAMK4	−5.90	2.6 × 10^−8^	1.5 × 10^−6^
CAPG	3.73	4.0 × 10^−10^	5.5 × 10^−7^	CLEC1B	−22.88	6.8 × 10^−5^	3.7 × 10^−2^
CBX8	2.73	1.9 × 10^−15^	9.0 × 10^−13^	DNASE1L3	−6.63	1.4 × 10^−5^	5.2 × 10^−3^
CCL14	−2.93	2.2 × 10^−7^	5.4 × 10^−4^	SOCS2	−7.10	2.0 × 10^−9^	6.9 × 10^−8^
CD226	−3.03	8.9 × 10^−9^	1.6 × 10^−5^	UBE2S	5.93	1.4 × 10^−5^	5.2 × 10^−3^
CKLF	2.71	1.1 × 10^−11^	1.1 × 10^−8^	VIPR1	−12.77	5.2 × 10^−12^	6.3 × 10^−11^
CLEC1B	−26.29	7.3 × 10^−12^	6.9 × 10^−9^				
CYP2C9	−3.34	7.8 × 10^−8^	1.7 × 10^−4^				
DNASE1L3	−6.62	2.2 × 10^−13^	1.6 × 10^−10^				
IGKV4-1	−5.33	1.7 × 10^−6^	4.9 × 10^−3^				
IMPDH1	2.08	3.6 × 10^−6^	1.2 × 10^−2^				
IQGAP2	−1.97	1.3 × 10^−9^	1.9 × 10^−6^				
KITLG	2.14	4.6 × 10^−6^	1.5 × 10^−2^				
KLRD1	−2.54	7.3 × 10^−7^	2.0 × 10^−3^				
LPCAT1	3.07	1.2 × 10^−10^	1.4 × 10^−7^				
MSC	6.00	2.1 × 10^−10^	2.6 × 10^−7^				
NDRG2	−2.46	1.2 × 10^−9^	1.7 × 10^−6^				
PGF	2.06	5.2 × 10^−6^	1.7 × 10^−2^				
RAB24	2.31	9.6 × 10^−15^	5.1 × 10^−12^				
SEC61G	2.14	5.1 × 10^−9^	8.7 × 10^−6^				
SOCS2	−6.45	5.3 × 10^−19^	1.3 × 10^−16^				
SPP1	25.97	8.7 × 10^−22^	1.3 × 10^−19^				
TAGAP	−2.49	9.6 × 10^−6^	3.3 × 10^−2^				
UBE2S	5.14	2.2 × 10^−21^	3.7 × 10^−19^				
VIPR1	−10.80	6.2 × 10^−27^	4.0 × 10^−25^				
**HCC Drinkers with HBV vs. Drinker Normals**				**HCC Drinkers vs. HCC Nondrinkers**			
**Gene Name**	**Fold Change**	**FDR**	***p*** **-Value**	**Gene Name**	**Fold Change**	**FDR**	***p*** **-Value**
APLN	18.60	2.6 × 10^−6^	5.0 × 10^−4^	CCL18	1.72	1.0 × 10^−2^	2.6 × 10^−2^
CAMK4	−3.83	4.4 × 10^−5^	1.7 × 10^−2^	CKLF	1.36	3.5 × 10^−3^	5.1 × 10^−3^
CLEC1B	−28.26	7.9 × 10^−8^	5.2 × 10^−6^	MMP9	1.88	5.3 × 10^−3^	1.0 × 10^−2^
DNASE1L3	−5.45	9.6 × 10^−6^	2.6 × 10^−3^				
NDRG2	−2.97	3.9 × 10^−7^	4.2 × 10^−5^				
UBE2S	4.85	1.0 × 10^−4^	4.8 × 10^−2^				
VIPR1	−8.65	2.1 × 10^−7^	1.9 × 10^−5^				
**HCC Drinkers with HBV vs. Nondrinker Normals**				**HCC Nondrinkers with HBV vs. Nondrinker Normals**			
**Gene Name**	**Fold Change**	**FDR**	***p*** **-Value**	**Gene Name**	**Fold Change**	**FDR**	***p*** **-Value**
CAMK4	−2.71	5.7 × 10^−9^	8.0 × 10^−6^	APLN	13.78	1.2 × 10^−33^	1.6 × 10^−31^
CAPG	3.47	3.1 × 10^−9^	4.2 × 10^−6^	CAMK4	−2.89	9.0 × 10^−13^	1.3 × 10^−9^
CBX8	2.23	3.6 × 10^−12^	2.5 × 10^−9^	CAPG	3.30	1.1 × 10^−9^	2.7 × 10^−6^
CCL14	−3.01	2.8 × 10^−9^	3.7 × 10^−6^	CBX8	2.14	7.7 × 10^−14^	9.9 × 10^−11^
CD226	−2.39	1.0 × 10^−6^	2.4 × 10^−3^	CCL14	−3.76	4.7 × 10^−20^	2.7 × 10^−17^
CKLF	3.26	6.9 × 10^−14^	3.3 × 10^−11^	CD226	−2.82	1.1 × 10^−11^	1.9 × 10^−8^
CLEC1B	−32.63	7.6 × 10^−20^	1.1 × 10^−17^	CLEC1B	−76.69	7.5 × 10^−45^	3.9 × 10^−43^
CYP2C9	−3.38	2.7 × 10^−7^	5.7 × 10^−4^	CTHRC1	13.81	3.5 × 10^−19^	2.2 × 10^−16^
DNASE1L3	−5.40	4.4 × 10^−14^	2.0 × 10^−11^	CYP2C9	−3.88	3.3 × 10^−12^	5.3 × 10^−9^
DUSP10	−2.15	7.5 × 10^−8^	1.4 × 10^−4^	DNASE1L3	−7.82	1.7 × 10^−37^	1.6 × 10^−35^
KITLG	2.97	1.2 × 10^−11^	9.7 × 10^−9^	DUSP10	−2.35	5.0 × 10^−14^	6.2 × 10^−11^
KLRD1	−2.73	1.0 × 10^−9^	1.2 × 10^−6^	FYN	−2.03	1.5 × 10^−12^	2.3 × 10^−9^
LPCAT1	2.76	2.1 × 10^−9^	2.6 × 10^−6^	KITLG	2.39	7.4 × 10^−8^	2.3 × 10^−4^
MSC	7.90	2.4 × 10^−11^	2.1 × 10^−8^	KLRD1	−2.82	1.3 × 10^−11^	2.2 × 10^−8^
NDRG2	−2.79	4.8 × 10^−19^	8.3 × 10^−17^	LPCAT1	2.16	7.6 × 10^−8^	2.4 × 10^−4^
RAB24	2.26	5.0 × 10^−14^	2.3 × 10^−11^	MSC	6.65	8.2 × 10^−9^	2.2 × 10^−5^
SOCS2	−3.70	1.6 × 10^−9^	2.0 × 10^−6^	MT-RNR2	−2.21	1.5 × 10^−11^	2.7 × 10^−8^
SPP1	15.71	6.9 × 10^−15^	2.7 × 10^−12^	NDRG2	−2.81	7.8 × 10^−23^	3.2 × 10^−20^
UBE2S	4.06	2.2 × 10^−17^	5.5 × 10^−15^	PGF	3.09	5.0 × 10^−8^	1.5 × 10^−4^
VIPR1	−7.31	1.2 × 10^−16^	3.5 × 10^−14^	RAB24	2.19	1.3 × 10^−17^	1.0 × 10^−14^
				SOCS2	−3.93	3.9 × 10^−16^	3.7 × 10^−13^
				TNFRSF4	5.61	9.0 × 10^−27^	2.3 × 10^−24^
				UBE2S	3.67	6.8 × 10^−20^	4.0 × 10^−17^
				VIPR1	−13.83	9.5 × 10^−55^	1.3 × 10^−53^

**Table 2:** Differential mutation load of selected immune-associated pathways.

Comparison: HCC Drinkers vs. HCC Nondrinkers		
Pathway Name	Odds Ratio	*p*-Value
B-cell receptor pathway	2.735	0.004
T-cell receptor pathway	1.901	0.029
Classical complement system activation pathway	2.184	0.047

## Supplementary Materials

The following are available online at https://www.mdpi.com/2072-6694/11/9/1273/s1, Table S1: GSEA results for list of miRNAs associated with each IA gene, Table S2: List of potential miRNA-mRNA interactions as defined by GSEA leading-edge subsets.
